# Social‐ecological theory, substance misuse, adverse childhood experiences, and adolescent suicidal ideation: Applications for community–academic partnerships

**DOI:** 10.1002/jcop.22560

**Published:** 2021-05-04

**Authors:** Semra A. Aytur, Sydney Carlino, Felicity Bernard, Kelsi West, Victoria Dobrzycki, Riana Malik

**Affiliations:** ^1^ Department of Health Management and Policy University of New Hampshire Durham New Hampshire USA; ^2^ Institute for Health Practice and Policy (IHPP), College of Health and Human Services University of New Hampshire Concord New Hampshire USA

**Keywords:** adverse childhood experiences (ACEs), food insecurity, mental health, nutrition, physical activity, policy, resilience, socioecological model

## Abstract

Suicide is the second leading cause of death among youth in the United States. Data from the 2015 Youth Risk Behavior Survey of 9th–12th grade students in New Hampshire (*N* = 14,837) were utilized. Adjusted odds ratios (aORs) and 95% confidence intervals (CIs) were estimated using logistic regression models to evaluate associations between suicidal ideation, adverse childhood experiences (ACEs), and other risk factors including using opioids/drugs without a prescription and food insecurity. We also examined whether potentially protective behaviors may attenuate the relationship between ACEs and suicidal ideation. The prevalence of suicidal ideation was 15.4% (girls 20.15; boys 10.67). In unadjusted models, the crude odds ratio reflecting the relationship between suicidal ideation and higher ACE scores was 1.85 (95% CI 1.76–1.94). In adjusted models, suicidal ideation remained positively associated with higher ACE scores (aOR 1.61, 95% CI 1.52–1.70). Risk and protective behavioral factors identified in relation to suicidal ideation and ACEs are discussed within the context of community–academic partnerships and policy.

## 1 INTRODUCTION

Suicide is the second leading cause of death among individuals aged 10–34 in the United States (National Institute of Mental Health [NIMH], 2019). The Social‐Ecological Model posits that health is influenced by interrelated factors at the individual, interpersonal, organizational, environmental, and policy levels (Crosby et al., 2013; Max et al., 2015). This perspective encourages multisectoral collaboration and emphasizes the use of data for translating research into practice and policy. The aim of this study was to evaluate associations between suicidal ideation (seriously considering attempting suicide within the past 12 months), adverse childhood experiences (ACEs; which include violence (e.g., physical bullying, cyber‐bullying, forced sexual activity, having a parent that is incarcerated, abusive, or has substance misuse issues; Dube et al., 2003), and other risk factors such as opioid/prescription drug use, food insecurity, and poor nutrition (proxied by high consumption of sugar‐sweetened beverages). We also examined whether potentially protective factors (such as having a parent or adult to talk to, participating in community activities, and getting recommended levels of physical activity) may attenuate relationships between ACE scores, risk factors, and suicidal ideation.

In New Hampshire, multisectoral partners including hospitals, schools, community‐based organizations, universities, and public health networks are working collaboratively to reduce suicide, address substance misuse, and improve mental health. These groups require data to enable them to better understand modifiable risk and protective factors that may buffer against suicidal ideation. We utilized data from the New Hampshire Youth Risk Behavior Surveillance System (YRBSS; Centers for Disease Control and Prevention [CDC], 2018; New Hampshire Department of Health and Human Services [DHHS], 2015) a cross‐sectional public health surveillance survey, to support community–academic partnerships and inform suicide prevention interventions. Although the state Department of Health and Human Services and the Department of Education report basic prevalence data and trend data from the YRBSS to guide these efforts, academic partners can offer more specific analytic expertise and statistical modeling to inform program planning, practice, policy development, and evaluation. Academic–community partnerships also enable students to learn quantitative, qualitative, and communicative skills that support interprofessional teams that can respond to the real‐world needs of communities. Because New Hampshire has witnessed increasing trends in suicide over the past decade, along with high rates of mortality from opioid overdoses, mental health, and substance misuse prevention have become top public health priorities. Furthermore, as communities around the world are struggling to address the mental health issues associated with the COVID‐19 pandemic (Polizzi et al., 2020) place‐based, data‐driven initiatives are becoming increasingly important.

### 1.1 Adverse childhood experiences

ACEs are traumatic events in the first 18 years of life such as abuse, neglect, partner violence, parent violence, and substance abuse in the home, divorce, and mental illness in the home (Substance Abuse and Mental Health Services Administration [SAMHSA], 2020). The foundational work on ACEs originated from the ACE Study, a large epidemiologic study among adult health maintenance organization members that was designed to assess the impact of childhood stressors (i.e., abuse, neglect, and other forms of family dysfunction) on a wide range of health behaviors and outcomes in adolescence and adulthood (Felitti et al., 1998; Scheel‐Jones, 2013). Subsequent studies have shown that traumatic and stressful childhood events increase the likelihood of multiple adolescent risk behaviors, including alcohol misuse, tobacco use, drug use, sleep deprivation, risky sexual behaviors, as well as other unhealthy behaviors (Anda et al., 2006; Campbell et al., 2016; Dube et al., 2001; Ramiro et al., 2010; Rothman et al., 2008).

Prior research suggests that 38% of children in every state of the United States have experienced at least one ACE (Robert Wood Johnson Foundation, 2017; Scheel‐Jones, 2013). These ACEs can range from death or incarceration of a parent/guardian, seeing or being a victim of abuse, and living with someone who has been suicidal (Anda et al., 2006; Robert Wood Johnson Foundation, 2017; Scheel‐Jones, 2013). These experiences may also include physical, emotional, and sexual abuse, neglect (physical and emotional), and household dysfunction including mental illness, incarcerated relatives, mother treated violently, substance abuse, and divorce.

A report by SAMHSA (2020) documented that 28% of respondents reported physical abuse and 21% reported sexual abuse. Additionally, other ACEs that are common are children experiencing divorce or having a parent or guardian struggle with substance abuse. ACEs are also known to cluster, meaning many children experience one or more adverse experiences (Wiens et al., 2020). For example, 40% of the U.S. population reported two or more ACEs and 12.5% reported four or more.

International research suggests that similar patterns exist in developing countries (Ramiro et al., 2010; Salawu & Owoaje, 2014). For research purposes, ACEs can be summed to derive an “ACE Score” that represents the different types of traumatic experiences that an individual has been exposed to (Wiens et al., 2020). Analyses conducted by researchers at the Robert Wood Johnson Foundation (2017) showed that 40% of children who are White have reported having one or more ACE compared with 51% for Hispanic children and 64% of children who are Black. Additionally, lower‐income families tend to have a higher prevalence of ACEs. Sixty‐two percent of children whose family income was 200% under the federal poverty level had one or more ACE. However, ACEs are observed across all income levels.

### 1.2 ACEs, substance misuse, and mental health

Previous research has linked ACEs to lifelong challenges including disability, increased risk of violence and victimization, mental health issues, substance misuse, and death (Clayton et al., 2019; Felitti et al. 1998; Dube et al., 2001; Anda et al., 2006). Schilling et al. (2007) examined self‐reported lifetime exposure to a range of ACEs in a community sample of high school seniors to depressive symptoms, drug abuse, and antisocial behavior during the transition to adulthood. The authors found that ACEs were strongly associated with all three outcomes. These results are important because rates of suicide and substance misuse are increasing in the U.S. Approximately one in seven young adults aged 18–25 years have a substance use disorder and prior research suggests that substance misuse is a major risk factor for suicide (Wang & Yen, 2017; Wolitzky‐Taylor et al., 2010; SAMHSA, 2019). Prior research also suggests that 30% of suicide deaths involve opiates (including heroin and prescription painkillers) and 75% of suicides involve one or more substances (CDC, 2018). ACE scores have shown strong relationships with initiating alcohol and drug use at a young age (National Institute of Drug Abuse (NIDA), https://www.drugabuse.gov/drugs-abuse/opioids; Rothman et al., 2008; Salawu & Owoaje, 2014; SAMHSA, 2020). Specifically, childhood abuse and parental drug use in the home increase a child's risk of misusing substances (American Society of Addiction Medicine, 2016; NIDA, https://www.drugabuse.gov/drugs-abuse/opioids). Furthermore, for every additional ACE, the number of prescription drugs used increased by 62% (SAMHSA, 2020). Forster et al. (2017) found that roughly 3% of students reported using prescription medication without a prescription and the majority also had at least one ACE.

Since the 1990s the United States has been experiencing high rates of opioid use and abuse (NIDA, https://www.drugabuse.gov/drugs-abuse/opioids; American Society of Addiction Medicine, 2016). NIDA defines opioids as, “a class of drugs that include the illegal drug heroin, as well as pain relievers available by prescription—oxycodone, hydrocodone, Vicodin, codeine, morphine, fentanyl, and many others” (NIDA, https://www.drugabuse.gov/drugs-abuse/opioids). The opioid crisis in the United States escalated in the 1990s when prescription opioids were being used to address the problem of undertreating people who suffered from pain. Drugs that are commonly prescribed to treat pain include oxycodone (OxyContin), hydrocodone (Vicodin), morphine, and methadone. In 2015, two million Americans were addicted to prescriptions. When assessing prescription drug misuse among youth nationally, YRBSS data suggest that 10% of White, 13% of Hispanic, and 12% of Black have reported misusing prescription drugs in their lives (CDC, 2018). Additionally, a 15‐year study found that hospitalizations related to prescription opioid overdoses among youth have almost doubled (Gaither et al., 2018).

New Hampshire, like many other states, is struggling to build capacity to address the inter‐connected issues of prescription opioid misuse, suicide, and ACEs. New Hampshire ranks 5th in the United States for opioid‐related deaths (American Society of Addiction Medicine, 2016). A study conducted in 2019 showed that New Hampshire had the largest increase in the rate of opioid‐deaths of people between 25 and 64 (Woolf & Schoomaker, 2019). The authors reported that the opioid‐related mortality rate among young and middle‐aged people in New Hampshire increased by 23% between 2010 and 2017. New Hampshire's suicide rates are also 50% higher than the national average (NAMI New Hampshire State Suicide Prevention Council SPC and Youth Suicide Prevention Assembly YSPA, 2018). Suicide is the second leading cause of death for individuals under 24‐years old in New Hampshire. In 2017, suicide deaths were the highest in two decades among young people.

**Figure 1:**
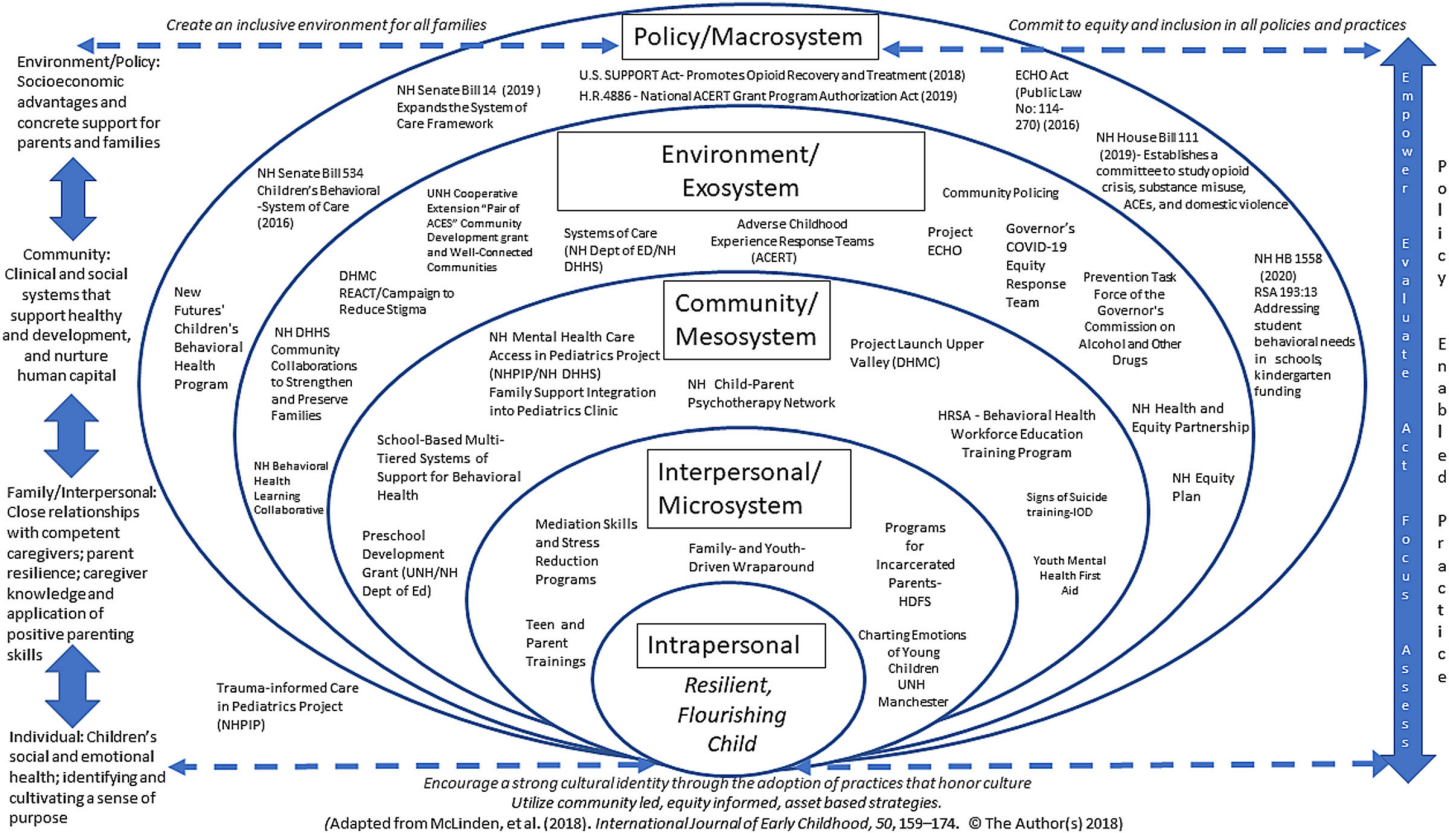
Conceptual model

These statistics have been accelerating the impetus for mobilizing multisectoral partnerships. Utilizing public health surveillance data to examine ACEs in relation to prescription drug misuse and other risks/protective factors among New Hampshire high school students allows these issues to be considered in a more integrated manner. For example, the “Take Action Cycle” (Robert Wood Johnson Foundation, 2020) focuses on the following iterative steps: (1) Assess needs and resources; (2) focus on what is important to communities; (3) choose effective policies and programs; (4) act on what is important; and (5) evaluate actions. Figure 1 illustrates how the “Take Action Cycle” iteratively informs layers of the Social‐Ecological Model with particular relevance to ACEs in New Hampshire. Similar action cycles have been described in the implementation science literature (Field et al., 2014; Kwan et al., 2019; McLinden et al., 2018).

### 1.3 Theoretical frameworks

Researchers have identified factors that may help to reduce the long‐term negative health impacts associated with ACEs and substance misuse (Forster et al., 2017; David‐Ferdon et al., 2016). These factors may operate at various levels of the Social‐Ecological Model, including the family, community, environment, and policy levels. Figure 1 illustrates our conceptual model, which connects Bronfrenbrenner's (1979) ecological systems theory and the Social‐Ecological Model to the “Take Action Cycle” for ACEs.

Notably, Bronfenbrenner's theory focuses on the interactions among processes, persons, contexts, and time from a human development perspective. Bronfenbrenner posited that these interactions were situated within four systems that shaped a child's development: The microsystem, the mesosystem, the exosystem, and the macrosystem. These four systems align with the levels of the Social‐Ecological Model, as shown in Figure 1. Our model echoes Bronfenbrenner's theory from a community development perspective, showing how multisectoral partnerships, programs, and policies evolve over time across levels of the Social‐Ecological Model to support a culture of health for children and families. This aligns with current conceptualizations such as the “Pair of ACEs” tree (Ellis & Dietz, 2017) and international bioecological systems models (McLinden et al., 2018) in which community resources relating to the social determinants of health (e.g., access to healthy food, housing, safe recreational spaces, transportation, educational opportunities, and clinical and social services) affect the mental and emotional resources of children and families.

Equity components drawn from the New Hampshire COVID‐19 Equity Taskforce recommendations (NH DHHS, 2020a) and Srivastav et al. (2020) have been added to our conceptual model, using dotted lines (Figure 1), to illustrate the relative fragility of actions pertaining to equity and the need for greater commitment to these components.

Using our conceptual model as a guide, the present study provides an analysis illustrating how public health surveillance data can inform the “assessment” step of the “Take Action Cycle.” This helps to facilitate the selection of effective, equitable programs and policies while building a foundation for ongoing evaluation over time.

### 1.4 Hypothesis

Using the conceptual model as a guide, we hypothesized that (1) ACEs would increase the odds of suicidal ideation and (2) protective factors (such as having a parent or adult to talk to, participating in community activities, and achieving recommended levels of physical activity) would attenuate associations between ACE scores and suicidal ideation.

## 2 METHODS

Data from the 2015 New Hampshire YRBSS, a cross‐sectional survey of 9th‐ to 12th‐grade students (*N* = 14,837), were analyzed. Developed in 1990, the YRBSS monitors health behaviors that contribute to the leading causes of death, disability, and social problems among youth and adolescents in the U.S. (CDC, 2018). This survey is conducted nationwide in U.S. schools, among high school students in 9th–12th grade. The YRBS is conducted every 2 years. Details pertaining to the complex sampling strategy are published by CDC (2015). Self‐reported ACEs were summed to create an ACE score that ranged from 0 to 8, based on methods used in prior studies (Campbell et al., 2016; Dube et al., 2006; Scheel‐Jones, 2013).

### 2.1 Measures

Questions included in the YRBS can be found in the New Hampshire YRBS 2015 Codebook (NH DHHS, 2015); those relevant to our analysis are defined below:

#### 2.1.1 ACE score

ACEs are traumatic events in the first 18 years of life such as abuse, neglect, partner violence, parent violence, and substance abuse in the home, divorce, and mental illness in the home. For the purposes of this study, we used eight questions from the NH YRBS that related to ACES:

Q21‐Have you ever been physically forced to have sexual intercourse when you did not want to?

Q22‐During the past 12 months, how many times did someone you were dating or going out with physically hurt you on purpose?

Q23‐During the past 12 months, how many times did someone you were dating or going out with, force you to do sexual things that you did not want to do?

Q24‐During the past 12 months, have you ever been bullied on school property?

Q25‐During the past 12 months, have you ever been electronically bullied?

Q92‐Have you ever seen or heard adults in your home slap, hit, kick, punch, or hurt each other?

Q101‐During the past 12 months, have either of your parents or other adults in your family been in jail or in prison?

Q104‐Have you ever lived with anyone who had a problem with alcohol or drugs?

#### 2.1.2 Suicidal ideation

We used the following question from the YRBS: During the past 12 months, did you ever seriously consider attempting suicide? (Coded as 1 [*Yes*], 0 [*No*]).

#### 2.1.3 Opioid/Prescription drug misuse

We used the following question from the YRBS: During your life, how many times have you taken a prescription drug (such as OxyContin, Percocet, Vicodin, codeine, Adderall, Ritalin, or Xanax) without a doctor's prescription? (coded as 0 [*never*] or 1 [*any use*]).

#### 2.1.4 Food insecurity

How often did you go hungry because there was not enough food in your home? (coded on a scale of 0 [*Never*] to 4 [*Always*]).

#### 2.1.5 Physical activity

During the past 7 days, on how many days were you physically active for a total of at least 60 min/day? (Coded as 1 if physically active 7 days/week, as recommended by the CDC; 0 if fewer than 7 days).

#### 2.1.6 Perception that drugs are easy to get

If you wanted to get a prescription drug (such as OxyContin, Percocet, Vicodin, codeine, Adderall, Ritalin, or Xanax) without a doctor's prescription, how hard or easy would it be for you to get some? (Coded as 1 [*Very hard*]; 2 [*Sort of hard*]; 3 [*Sort of easy*]; 4 [*Very easy*]).

#### 2.1.7 High soda consumption

During the past 7 days, how many times did you drink a can, bottle, or glass of soda or pop, such as Coke, Pepsi, or Sprite? (coded as 1 [*at least once per day*], 0 [*less than once per day*]).

#### 2.1.8 Community service

During the past 30 days, how many times did you perform any organized community service as a nonpaid volunteer, such as serving meals to the elderly, picking up litter, helping out at a hospital, or building homes for the poor? (coded as 1 [*at least once*], 0 [*none*]).

#### 2.1.9 Parent support

When you feel sad, empty, hopeless, angry, or anxious, with whom would you most likely talk about it? (Coded as 1 if the respondent reported having a parent, adult family member, or other adults to talk to; 0 otherwise).

#### 2.1.10 Grades in school

During the past 12 months, how would you describe your grades in school? (Coded on a scale from 1 [*mostly F's*] to 5 [*mostly A's*]).

### 2.2 Analysis

**Figure 2:**
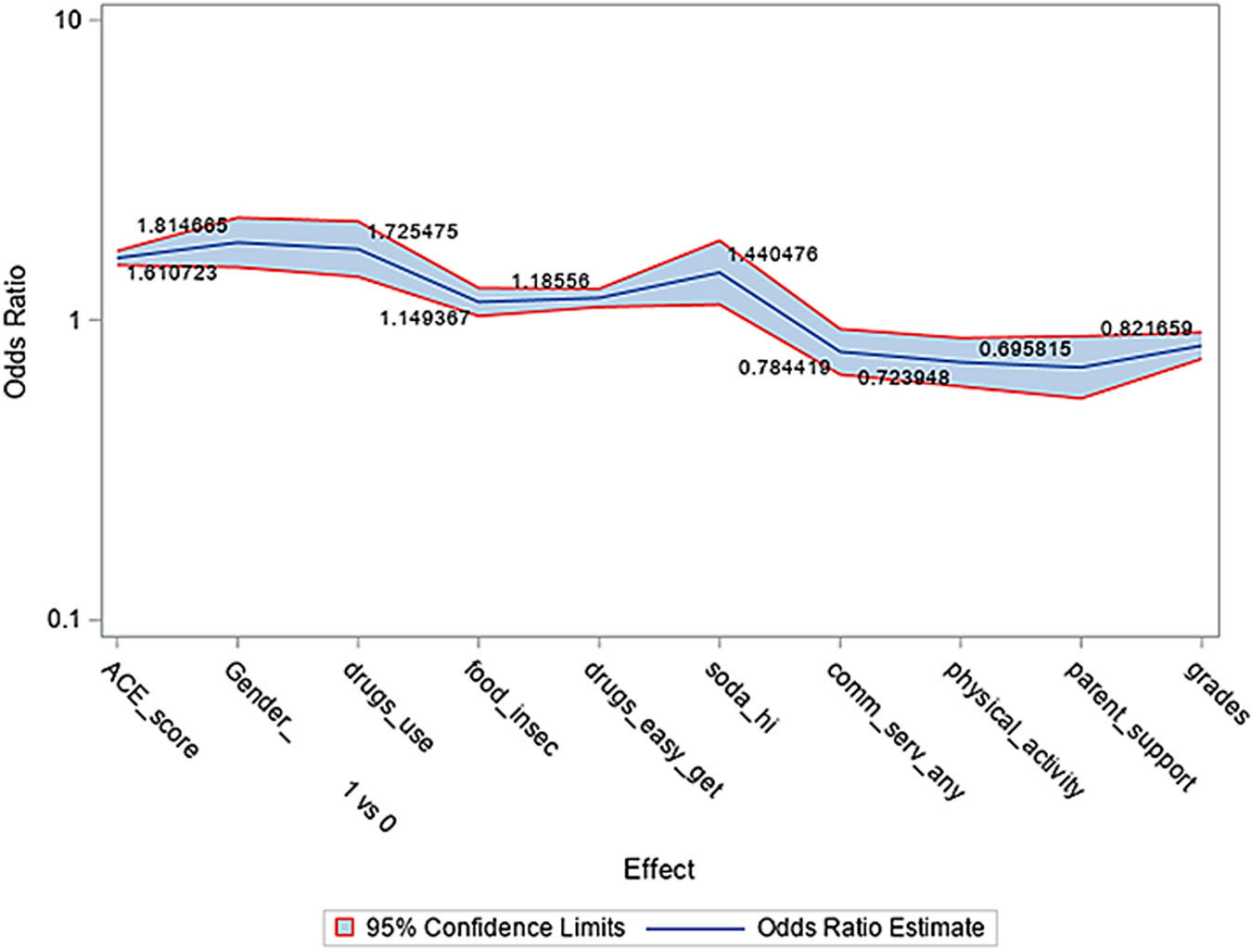
Graphical depiction of odds ratios for adverse childhood experience (ACE) scores and risk/protective factors, with respect to suicidal ideation among youth (*N* = 10,603)

Descriptive statistics (e.g., univariate analyses, percentages) were calculated using appropriate sample weights (Appendix A). The CDC employs a three‐stage cluster sampling strategy stratified by racial/ethnic concentration and MSA (metropolitan statistical area) status (CDC, 2015). Overall weights are scaled so that the weighted count of students equals the total sample size, and the weighted proportions of students in each grade match the national population proportions. Thus, the data are representative of students in grades 9–12 in public and private schools in the state. Guided by our conceptual model, the following model‐building process was employed to evaluate associations between suicidal ideation (seriously considering attempting suicide within the past 12 months) and ACE scores, as follows: Model 1 assessed the crude (unadjusted) association between suicidal ideation and ACE scores; Model 2 assessed this association while controlling for sociodemographics (age, race, gender, grade in school); and Model 3 assessed the association while controlling for sociodemographics plus other risk/protective factors (e.g., opioid/prescription drug use, perceived ease of obtaining opioid/prescription drugs, food insecurity, consumption of sugar‐sweetened beverages, physical activity, community service, and having a trusted adult to talk to). The risk and protective (resilience‐oriented) factors selected for potential inclusion were chosen a priori, based on the theoretical framework, review of prior literature, and stakeholder knowledge (e.g., Cooperative Extension workgroups, clinical partners, and public health networks). The final model (Figure 2) represented the best‐fit model in which several covariates that were not statistically significant (e.g., race and age) were removed. Analyses were conducted using SAS statistical software v.9.4 (SAS Institute Inc., 2019), and accounted for the complex survey‐sampling design (CDC, 2015).

## 3 RESULTS

Overall, the prevalence of suicidal ideation was 15.4% (girls, 20.1%; boys 10.7%) in 2015. Adverse childhood experiences, opioid/prescription drug use, and food insecurity/nutrition emerged as significant risk factors for suicidal ideation among New Hampshire youth. The ACE score ranged from 0 to 8, with a median of 1.0 and an interquartile range of 2.0. The three most frequently reported ACEs were living with a person who has a problem with alcohol or drugs (32.1%), having been bullied on school property over the past 12 months (24.1%), and having been electronically bullied over the past 12 months (19.3%). The prevalence of specific ACEs varied by gender. For example, 9.8% of girls answered affirmatively to the question “Have you ever been physically forced to have sexual intercourse when you did not want to?” compared to 3.1% of boys. Twelve percent of girls reported having been forced to “do sexual things that you did not want to do” with someone they were dating or going out with, compared to 4% of boys. Bullying on school property was reported by 27.4% of girls, compared to 16.8% of boys; electronic bullying was reported by 26.1% of girls and 11.4% of boys (nonbinary gender identity questions were not available for this analysis).

With regard to other risk factors, 14.7% of youth reported having taken a prescription drug (such as OxyContin, Percocet, Vicodin, codeine, Adderall, Ritalin, or Xanax) without a doctor's prescription, 24% reported ever going hungry because there was not enough food in the home, 24% reported that opioid/prescription drugs were “somewhat easy” or “very easy” to get, and 13.1% reported drinking soda at least once per day or more. Twenty‐two percent of youth reported meeting the CDC's recommendation of being physically active 7 days/week for a total of at least 60 min/day, and 42.6% reported performing any organized community service (such as serving meals to the elderly, picking up litter, helping out at a hospital, or building homes for the poor) over the past 30 days. Seventeen percent reported that they had a parent, adult family member, or another adult to talk to when they feel sad, empty, hopeless, angry, or anxious.

In unadjusted logistic regression models, the crude odds ratio reflecting the relationship between suicidal ideation and higher ACE scores was 1.85 (95% confidence interval [CI] 1.76–1.94). In adjusted models, suicidal ideation remained positively associated with higher ACE scores (aOR 1.61, 95% CI 1.52–1.70) (Table 1). Other factors that were positively associated with suicidal ideation included food insecurity, high soda consumption, use of opioid/prescription drugs without a prescription, and perceptions that drugs were easy to get. Protective factors that were negatively associated with suicidal ideation included having a parent or other adult to talk to, being involved in community service, participating in recommended levels of physical activity (7 days/week for at least 60 min), and academic achievement (Figure 2). These factors attenuated the effect of ACEs.

**Table 1:** Associations between suicidal ideation, adverse childhood experiences (ACE) score, and other risk and protective factors among New Hampshire youth

Odds ratio estimates from multivariable logistic regression models (*N* = 10,603)				
Effect	Point estimate	95% confidence limits		*p* Value
ACE score	1.611	1.525	1.702	<0.0001
Gender—1 versus 0 (female vs. male)	1.815	1.499	2.197	<0.0001
Drug use	1.725	1.393	2.137	<0.0001
Food insecurity	1.149	1.031	1.281	0.0121
Drugs easy to get	1.186	1.105	1.272	<0.0001
High soda consumption	1.440	1.126	1.843	0.0037
Community service	0.784	0.658	0.935	0.0068
Physical activity	0.724	0.600	0.873	0.0007
Parent support	0.696	0.547	0.885	0.0031
Grades in school	0.822	0.742	0.91	0.0002

## 4 DISCUSSION

The present study applied social‐ecological theory to examine ACEs in relation to other risk and protective factors among adolescents in New Hampshire. Overall, our results support those of other researchers. For example, Thompson et al. (2012) examined the association between recent ACEs and suicidal ideation in a sample of 740 at‐risk adolescents. The authors found that ACEs were positively associated with suicidal ideation and that recent physical abuse and psychological maltreatment were uniquely associated with suicidal ideation. In a study examining the relationship of ACEs to alcohol use, Dube et al. (2006) found that the total number of ACEs (ACE score) demonstrated a strong dose–response relationship with respect to initiating alcohol use during early adolescence.

These findings align with the results of our study, which suggest that a one‐unit increase in the ACE score is associated with 1.6 times higher odds of suicidal ideation. Secondary analysis suggests that a one‐unit increase in the ACE score is associated with 1.4 times higher odds of using opioids and related drugs without a prescription. This suggests that an individual with five or more ACEs has eight times the risk of suicidal ideation and seven times the risk of using opioids without a prescription, controlling for other covariates. International research has revealed similar relationships between ACEs and risk behaviors. In Nigeria, for example, Salawu and Owoaje (2014) conducted a study of ACEs and observed a strong association between ACEs and smoking among urban youth.

### 4.1 Protective factors

An emerging body of research is also beginning to specifically explore ACEs and protective factors in adolescents (Danese & McEwen, 2012; Forster et al., 2017; Oral et al., 2016). The complexity of ACEs necessitates consideration of multidirectional relationships across different levels of the Social‐Ecological Model. For example, at the interpersonal level, prior research suggests that having a positive relationship with a parent or adult helped to decrease the association between adverse childhood experiences and nonmedical use of prescription medications (Forster et al., 2017). At the community level, prior research suggests that many people who are addicted to opioids are incarcerated and do not receive the treatment that they need (Global Commission on Drugs, 2017). The Illinois Criminal Justice Information Authority (ICJIA) conducted a study to examine substance abuse in the U.S., specifically focusing on youth (Reichert et al., 2017). The study concluded that youth who are at risk for substance misuse are those who have poor caregiver relationships, limited supervision, friends or family who misuse drugs or alcohol, academic underachievement, social skills that are not well developed, and easy access to drugs (Reichert et al., 2017). The ICJIA is an example of an organization that uses such data to make evidence‐informed recommendations regarding how to prevent and treat substance misuse in youth. Strategies recommended by the ICJIA include school‐based substance use prevention, which may include education programs on competency, social resistance, and normative education.

### 4.2 Using data to support the action cycle

In New Hampshire, there is increasing impetus to use data support community–academic partnerships around youth mental health, substance misuse, and other public health issues. Multisectoral partners are striving to build capacity to address these challenges through policy, systems, and environmental change (Sreedhara et al., 2017). For example, students, faculty, and staff at the University of New Hampshire have been using the YRBSS and related data over the past 5 years to help build the evidence base to support a system of care. Students from a variety of disciplines and their faculty mentors are working collaboratively to better understand the factors that increase vulnerability to suicidal ideation and substance misuse among the state's youth. They collaborated on efforts to support place‐based interventions, programs, and policies both within and outside the classroom. For example, instead of learning about statistics, policy, and research methods in a decontextualized manner, the students developed a passion for using data to tell a compelling story that can inform action. Since 2015, successive cohorts of students, faculty, and community partners have asked: “How can the community become a better support system for youth?”

**Figure 3:**
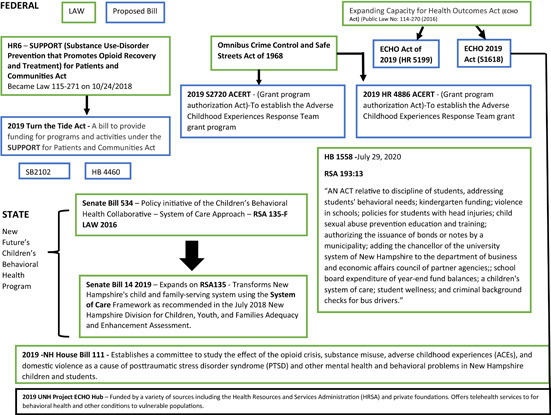
Policy examples

For example, results from the present study have been used to inform an evolving ecology of practices, programs, and policies in New Hampshire from 2015 to 2020 (Figure 1). Additional details specific to policy initiatives at state and federal levels are described in Figure 3, and descriptive information pertaining to various community‐level initiatives is provided in Table SA.

### 4.3 Reconnecting to social‐ecological theory and resilience

Over the past several decades, resilience theory (Folke et al., 2010; Holling, 1973) has emerged as a powerful tool for understanding the systems through which people interact with their environment via social‐ecological systems (Berkes et al., 2003; Ostrom, 2005, 2009). Resilience theory describes interactions between dynamic systems operating at a variety of spatial and temporal scales (Walker & Salt, 2006) which may accelerate changes in smaller subsystems. Within the broader domain of resilience theory, “social‐ecological resilience” refers to the ability of a social‐ecological system to absorb change and disturbance without shifting to a new state with a different set of processes and structures (i.e., without transforming into a new system state) (Clifton, 2010; Walker & Salt, 2006). Researchers have consistently documented the transformative adaptive capacity of both human and natural systems, which have implications for the ability of children to flourish (Bronfenbrenner, 1979; Brown & Williams, 2015; Walker & Salt, 2006). These lessons are particularly salient amid contemporary disruptions which include the COVID‐19 pandemic, climate change, violence/conflict, and systemic racism.

As specified by Bronfenbrenner's ecological systems theory (1979), the social, political, and the natural environment encompass a nested arrangement of structures in which children develop. Each structure is interdependent, and each can be influenced by collective action at various levels of the Social‐Ecological Model (Golden et al., 2015; Winch, 2012) (see Table SA). For example, intrapersonal factors including knowledge, skills, and self‐efficacy can be influenced by educational programs, public health communication campaigns, and appropriate clinical services. Interpersonal factors can be influenced by initiatives that target practices within families and peer groups. For example, a review conducted by the Robert Wood Johnson Foundation & Academy Health (2016) supports the use of interventions such as site‐based parent education programs, home visits, and concurrent treatment for substance abuse and parenting skills to mitigate the effects of ACEs among youth. In New Hampshire, an example of this type of intervention is the Trauma‐Informed Care in Pediatrics Project (NH Pediatric Improvement Partnership, 2020). Funded by New Hampshire Children's Health Foundation, this project aims to increase pediatric general practitioner knowledge about trauma‐informed care and existing tools to support addressing trauma in primary care settings. The project also provides support to four pediatric primary care clinics in using quality improvement principles to pilot process(es) to detect and respond to patients experiencing toxic stress.

Community factors include institutional arenas and norms that can influence both individual and collective choices. A tapestry of initiatives including the formation of Adverse Childhood Experience Response Teams (ACERTS) (New Hampshire Children's Health Foundation, 2020), campaigns to reduce stigma such as the REACT campaign (Dartmouth Hitchcock Medical Center [DHMC], 2020), community policing initiatives, and school‐based programs are being implemented in New Hampshire. For example, ACERTS are comprised of police officers, crisis services advocates, and behavioral health professionals. The teams are been trained to respond to incidents as soon as the scenes have been secured by police. The teams assess the situation and recommend appropriate interventions for the child such as support groups, mental health counseling, early childhood education, or child–parent psychotherapy.

Another integrative example that aims to improve outcomes at the individual, family, and community levels is Project LAUNCH (Linking Actions for Unmet Needs in Children's Health) (NH DHHS, 2020c). Project LAUNCH strives to promote positive behavioral health for New Hampshire's expectant families and children through age eight by focusing on building strong social and emotional skills in addition to physical and cognitive development. These examples rely primarily on grant‐funded initiatives that are woven together as the “Take Action Cycle” unfolds.

At the policy level, these actions are supported by a scaffolding of initiatives that support policy‐enabled practice (Embi et al., 2019) (see Figure 3 and Table SA).

For example, policies provide authorizing legislation and/or aim to enhance appropriations to strengthen a “systems of care” approach. In this manner, policies help to accelerate academic–community partnerships and interprofessional integration (NH DHHS, 2020b).

In May 2016, the New Hampshire Legislature passed Senate Bill 534‐FN which established the development of a comprehensive system of care for children's behavioral health services in the state. A recent report (New Hampshire Pediatric Improvement Partnership, 2020) outlined continued progress towards implementing a system of care for children. The report noted that there have been critical improvements to the children's behavioral health system of care, including “the expansion of the Medicaid to Schools program; funding for a new Medicaid benefit that allowed the DHHS to expand the provision of high fidelity wraparound and care coordination services to children with behavioral health needs (2018–2019 state budget); paving the way for further integration of high fidelity wraparound services; the inclusion of children in the 10‐year plan for mental health services; the expansion of the FAST Forward program; the continued development of school‐based behavioral health services through the Office of SAMHSA grants; the continued adoption of NH's Multi‐Tiered System of Supports for Behavioral Health and Wellness (MTSS‐B) Model by state school districts; and the expansion of school‐based prevention efforts including social‐emotional learning and a focus on school culture and climate” (New Hampshire Pediatric Improvement Partnership, 2020).

Another important bill, the New Hampshire House Bill (HB) 111 was passed into law in May 2019. This law establishes a committee to study the effect of the opioid crisis and domestic violence on children and will recommend legislation to address it. More recently, New Hampshire Senate Bill 282 was passed into law on August 20, 2019. The law mandates that schools provide training on recognizing the warning signs for suicide and effective prevention strategies for all staff members including teachers, food service employees, janitors, and volunteers. Schools will also be required to create policies for responding to teen suicides. An earlier effort to pass a bill requiring suicide prevention in schools failed in 2013. The effort was supported by multiple advocates, including community–academic partners and nonprofit organizations such as Connors Climb, which provides evidence‐based suicide prevention education to youth, schools, and communities.

Prior research also suggests that policies in other sectors are important in terms of mitigating the negative impacts of ACEs and substance misuse. For example, policies in the agricultural sector can support access to healthy foods (Lyn et al., 2013) and policies pertaining to land use and transportation planning that can help children to achieve recommended levels of physical activity (Aytur et al., 2016). New Hampshire's Cooperative Extension staff are playing an important role in facilitating academic–community partnerships in various policy and programmatic arenas including outdoor recreation, food access/nutrition, mental health, substance misuse, community economic development, and civic engagement.

### 4.4 Equity and inclusion

The tenets of equity and inclusion are central to our conceptual model (Figure 1). These tenets include recognizing the need to create an inclusive environment for all families, encouraging a strong cultural identity for all families, and demonstrating a commitment to equity and inclusion in all policies and practices (Annie E. Casey Foundation, 2015). The Empower Action Model (Srivastav et al., 2020) addresses childhood adversity as a root cause of disease by building resilience across multiple levels of influence to promote health, equity, and wellbeing. The model integrates evidence and blends important frameworks pertaining to ACEs including the Social‐Ecological Model, protective factors, inclusion, and the life course perspective. The authors also describe ways to use this model within community coalitions. One of the ways that these tenets are being operationalized in New Hampshire is through coalitions that comprise the Well‐Connected Communities (WCC) initiative (Sulzer, 2020). These coalitions utilize academic‐community partnerships to assess data‐driven needs and strengths in communities, as well as to promote equity and reduce health disparities. Within each WCC area, local health councils are developing and implementing action plans to address top public health priorities in their communities. Data is also being used to better understand gender‐related differences in ACEs, risk factors, and protective factors. This information may help to inform targeted interventions (Neger & Prinz, 2015; Cavanaugh et al., 2015; Oral et al., 2016) including new telehealth interventions via Project ECHO® (Ryer et al., 2020).

### 4.5 Limitations and directions for future research

Several limitations warrant mention. The cross‐sectional design of this study precludes causal inference. However, the data are useful for hypothesis generation and for raising awareness about youth mental health, substance misuse, and related risk and protective factors. A second limitation is that not every student answered every survey question, thus, there were missing data for some measures. Additionally, constructs such as nutritional status were crudely proxied by using available variables such as consumption of sugar‐sweetened beverages. Although consumption of sugar‐sweetened beverages has been shown to be correlated with poor nutritional status (Gan et al., 2019), it is not a sensitive or comprehensive measure. Additionally, as other researchers have noted, one of the challenges in studying ACEs is that the links and mechanisms between specific childhood experiences and their health outcomes have not been fully elucidated (Anda et al., 2006; Danese & McEwen, 2012). Hael et al. (2019) propose to build an integrated data set from multiple sources that could enable more effective ACE surveillance. This project, called SPACES, aims to provide an infrastructure to facilitate data sharing, integration, and improved surveillance of ACEs. More rigorous, randomized controlled trials are also needed to evaluate the most effective interventional approaches.

Our results show that ACEs significantly increase the odds of suicidal ideation among New Hampshire youth. However, other modifiable risk and protective factors are independently associated with suicidal ideation, even after controlling for ACEs. This study emphasizes the importance of considering interventions that span multiple levels of the Social‐Ecological Model to mitigate the impact of ACEs. Multisectoral interventions, policy changes, and community support systems are needed to buffer against suicidal ideation and build resilience. Such approaches may inform the community psychology/prevention science literature, facilitating a reduction in risky behavioral choices while protecting against future health issues in adulthood.

### PEER REVIEW

The peer review history for this article is available at https://publons.com/publon/10.1002/jcop.22560.

## Supporting information

Supporting information.

Supporting information.

## Data Availability

These data were derived from the YRBSS available in the public domain. The data that support the findings of this study are available from the CDC (YRBSS, 2015) at https://www.cdc.gov/healthyyouth/data/yrbs/index.htm and https://www.cdc.gov/nchhstp/dear_colleague/2016/dcl-060916-2015-youth-risk-behavior-survey-results-released.html

## APPENDIX A DESCRIPTIVE STATISTICS

**Table:**

Table of age					
Q1 (Age) (years)	Frequency	Weighted frequency	Standard error of weighted frequency	Percent	Standard error of percent
≤12	58	301.00	97.20985	0.5289	0.1718
13	12	40.00	12.72792	0.0703	0.0224
14	1,680	6530	780.94035	11.4732	1.2615
15	3,874	14,266	791.09027	25.0654	1.1081
16	3,846	14,011	678.71842	24.6174	1.0403
17	3,424	14,287	727.81404	25.1023	1.2854
≥18	1,768	7,480	518.50353	13.1424	0.8955
Total	14,662	56,915	1,485	100.0000	
Frequency missing = 175					

Table of gender					
Gender	Frequency	Weighted frequency	Standard error of weighted frequency	Percent	Standard error of percent
0	7,429	29,385	848.22955	51.8620	0.6187
1	7,175	27,275	792.63470	48.1380	0.6187
Total	14,604	56,660	1,483	100.0000	
Frequency missing = 233					

Table of grade_in_School					
Grade_in_School	Frequency	Weighted frequency	Standard error of weighted frequency	Percent	Standard error of percent
9	4,033	15,057	1,366	26.5748	2.1479
10	3,886	14,536	1,164	25.6552	1.9185
11	3,648	13,750	1,020	24.2680	1.7943
12	3,037	13,316	1,008	23.5020	1.7705
Total	14,604	56,659	1,482	100.0000	
Frequency missing = 233					

Table of race (White/non‐White)					
Race	Frequency	Weighted frequency	Standard error of weighted frequency	Percent	Standard error of percent
Non‐white	2,207	6,327	261.40035	10.9935	0.4381
White	12,630	51,225	1,414	89.0065	0.4381
Total	14,837	57,552	1,489	100.0000	

Table of ethnicity (Hispanic/non‐Hispanic)					
Hispanic	Frequency	Weighted frequency	Standard error of weighted frequency	Percent	Standard error of percent
Non‐Hispanic	13,635	53,524	1,463	94.8351	0.3377
Hispanic	877	2,915	183.91861	5.1649	0.3377
Total	14,512	56,439	1,473	100.0000	
Frequency missing = 325					

Table of ACE_score					
ACE_score	Frequency	Weighted frequency	Standard error of weighted frequency	Percent	Standard error of percent
0	5,470	23,594	689.27619	48.8752	0.8291
1	2,939	11,424	354.96108	23.6649	0.5218
2	1,800	7,057	335.85903	14.6186	0.5682
3	903	3,388	181.88462	7.0183	0.3093
4	463	1,800	146.07782	3.7287	0.2990
5	205	676.00000	60.95572	1.4003	0.1159
6	71	238.00000	32.90096	0.4930	0.0663
7	26	68.00000	14.90747	0.1409	0.0310
8	8	29.00000	10.63015	0.0601	0.0221
Total	11,885	48,274	1,177	100.0000	
Frequency missing = 2,952					

Table of suicidal ideation					
suic_ID	Frequency	Weighted frequency	Standard error of weighted frequency	Percent	Standard error of percent
0	11,771	47,961	1,183	84.6545	0.4432
1	2,379	8,694	319.41691	15.3455	0.4432
Total	14,150	56,655	1,354	100.0000	
Frequency missing = 687					
