# Supplementary material for: Social‐ecological theory, substance misuse, adverse childhood experiences, and adolescent suicidal ideation: Applications for community–academic partnerships
Source: J Community Psychol. 2021 May 4;50(1):265–84. doi: 10.1002/jcop.22560 (PMC9292564; doi:10.1002/jcop.22560)
Supplement: Supplementary file 1 — Supporting information. [file JCOP-50-265-s001.docx]

**Examples of Strategies to Address ACEs Using the Social-Ecological Model:**

**Policy Level**

**Federal Policies:**

**H.R.6** – **SUPPORT (Substance Use-Disorder Prevention that Promotes Opioid Recovery and Treatment) for Patients and Communities Act**

Sponsor: Rep. Walden, Greg [R-OR-2] (Introduced 06/13/2018)-

became public law 115-271on 10/24/2018.

**SB 2102**

Introduced in July 2019 partisan bill – democrat 3-0 “**Turn the Tide**” sponsors Sen. Jeanne Shaheen, Sen. Maggie Hassan and Sen. Chris Van Hollen

https://www.congress.gov/bill/116th-congress/senate-bill/2102/all-info

“A bill to provide funding for programs and activities under the SUPPORT for Patients and Communities Act”

**Same as H.R.4460 - Turn the Tide Act – Sponsor Ann Kuster (introduced Sept 2019)**

funds through FY2023 the SUPPORT for Patients and Communities Act, which established various programs to address opioid addiction (<https://www.congress.gov/bill/116th-congress/house-bill/4460>)

**S.2720** https://www.congress.gov/bill/116th-congress/senate-bill/2720/text – Jeanne Shaheen - **A bill to amend the Omnibus Crime Control and Safe Streets Act of 1968 to establish the Adverse Childhood Experiences Response Team grant program, and for other purposes**. October 2019 - referred to the Committee on the Judiciary - $10,000,000 for each of fiscal years 2020 through 2023 to fund grants to address adverse childhood experience in establishing protocols, developing referral partnership agreements, integrating law enforcement, mental health and crisis services, implement programs and practices, identify barriers to access, providing training to community partners and support cross-system planning and collaboration (same as **H.R.4886).**

**H.R.4886 - National ACERT Grant Program Authorization Act**

Sponsor: Rep. Pappas, Chris [D-NH-1] (Introduced 10/28/2019); Co-sponsor: Rep. Rutherford, John H. [R-FL-4]* and Rep. Kuster, Ann M. [D-NH-2]

House - 12/18/2019 Referred to the Subcommittee on Crime, Terrorism, and Homeland Security. To amend the **Omnibus Crime Control and Safe Streets Act of 1968** to establish the Adverse Childhood Experiences Response Team grant program, and for other purposes.

Link: <https://www.congress.gov/bill/116th-congress/house-bill/4886/text?r=5&s=1>

This Act may be cited as the “National ACERT Grant Program Authorization Act”.

 $10,000,000 for each of the fiscal years 2020 through 2023 (same as **S.2720)**

**Expanding Capacity for Health Outcomes Act or the ECHO Act** (Public Law No: 114-270 (12/14/2016))

This bill requires the Department of Health and Human Services (HHS) to report on technology-enabled collaborative learning and capacity building models, which connect specialists to primary care providers through videoconferencing to facilitate case-based learning, dissemination of best practices, and evaluation of outcomes. The report must include: (1) an analysis of the use, integration, and impact of such models; (2) a list of such models recently funded by HHS; (3) recommendations to reduce barriers to adoption of such models; (4) opportunities for adoption of such models into HHS programs; and (5) recommendations regarding the role of such models in continuing medical education.

Link: <https://www.congress.gov/bill/114th-congress/senate-bill/2873>

**State Policies:**

**New Futures' Children's Behavioral Health Program**

In 2016, New Hampshire passed Senate Bill 534, a major policy initiative of the Children's Behavioral Health Collaborative, which imbedded the system of care approach and accompanying values in RSA 135-F. The law requires the State to develop and maintain an integrated and comprehensive service delivery system for children with behavioral health needs. A System of Care is a behavioral health care approach that relies on a coordinated network of effective community-based services and supports with a broad array of individualized services which help children and youth to function better at home, in school, in the community, and throughout life.

**Senate Bill 14,** signed into law on June 3, 2019, expands and builds on RSA 135-F and years of work to build New Hampshire's System of Care for children's behavioral health. A major policy initiative of the New Hampshire Children's Behavioral Health Collaborative, Senate Bill 14 transforms New Hampshire's child and family-serving system using the System of Care Framework as recommended in the July 2018 New Hampshire Division for Children, Youth, and Families Adequacy and Enhancement Assessment.

The Law also requires the DOE and DHHS to produce yearly reports on the State's progress in developing a System of Care. Thus far, three reports have been produced:

**Systems of Care** (NH Dept of ED/NH DHHS)

Three-year report March 2019

<https://www.dhhs.nh.gov/dcbcs/bbh/documents/soc-year-3-report.pdf>

**2019 NH House Bill 111**

Establishes a committee to study the effect of the opioid crisis, substance misuse, adverse childhood experiences (ACEs), and domestic violence as a cause of posttraumatic stress disorder syndrome (PTSD) and other mental health and behavioral problems in New Hampshire children and students.

**Status:** (Passed) 2019-05-16 - Signed by Governor Sununu 05/15/2019; Chapter 19; Eff: 05/15/2019 [HB111 Detail]

**SPONSORS:** Rep. Mulligan, Graf. 12; Rep. Shurtleff, Merr. 11; Rep. Campion, Graf. 12; Rep. Walz, Merr. 23; Rep. Weber, Ches. 1; Rep. Heath, Hills. 14; Rep. Tanner, Sull. 9; Rep. Rice, Hills. 37; Rep. T. Smith, Hills. 17; Sen. Carson, Dist 14; Sen. Hennessey, Dist 5

**Committee members**: (a) Three members of the house of representatives, one of whom shall be from the health, human services and elderly affairs committee and one of whom shall be from the children and family law committee, appointed by the speaker of the house of representatives (b) One member of the senate, appointed by the president of the senate

Citation:

*NH HB111 | 2019 | Regular Session*. (2019, May 16). *LegiScan*. Retrieved November 09, 2020, from https://legiscan.com/NH/bill/HB111/2019

**NH Senate** **Bill 14** – In June of 2019, the NH Senate Bill 14 was passed. The bill focuses on child welfare and is broken down into five major categories.

Link:

<https://legiscan.com/NH/text/SB14/id/2012081/New_Hampshire-2019-SB14-Amended.html>

**NH House Bill 1558**

“AN ACT relative to discipline of students, addressing students' behavioral needs; kindergarten funding; violence in schools; policies for students with head injuries; child sexual abuse prevention education and training; authorizing the issuance of bonds or notes by a municipality; adding the chancellor of the university system of New Hampshire to the department of business and economic affairs council of partner agencies; change of school or assignment due to manifest educational hardship; school board expenditure of year-end fund balances; a children's system of care; student wellness; and criminal background checks for bus drivers.”

Link:

<http://gencourt.state.nh.us/bill_status/billText.aspx?sy=2020&id=1725&txtFormat=html>

**Community Level:**

**Adverse Childhood Experience Response Teams (ACERTS) in NH:**

Manchester

A partnership in Manchester has put together a response team that can be deployed to serve children who have been exposed to violence. The Adverse Childhood Experiences Response Team (ACERT) is made up of a police officer, a crisis services advocate, and a behavioral health professional. The team has been trained to respond to incidents as soon as the scenes have been secured by the police. The team will assess the situation and determine the next steps that could be taken for the child such as support groups, mental health counseling, early childhood education, or child-parent psychotherapy. <https://www.amoskeaghealth.org/adverse-childhood-experiences-response-team-acert/>

(The municipalities of Somersworth, Laconia, and Concord are also initiating ACERTS).

Citation:

# NH Children’s Health Foundation (2020). Case study: A Community Comes Together to Protect its Children. <https://nhchildrenshealthfoundation.org/acert-case-study/>

**Project ECHO (UNH)**

In 2019, the University of New Hampshire, as a new Project ECHO® Hub, launched its first program on medications for addiction treatment (Institute for Health Policy and Practice, 2020). The Project ECHO (Extension for Community Healthcare Outcomes) model was developed out of the University of New Mexico and focuses on reducing health disparities and demonopolizing knowledge across a variety of topics (The University of New Mexico, 2020b). Utilizing cased-based learning across a virtual learning platform, Project ECHO® applies an all teach, all learn approach to the guided practice model (The University of New Mexico, 2020a). The Project ECHO model has been used to discuss adverse childhood experiences across various ECHO hubs including Rutgers and the Center for Rural Health at the University of North Dakota School of Medicine & Health (Rutgers, 2019; Center for Rural Health, 2020). As a Project ECHO hub, the University of New Hampshire has launched multiple programs since 2019, including the NH Mental Health Care Access in Pediatrics (NH MCAP) ECHO through the NH Pediatric Improvement Partnership (NH PIP), which aims, “To increase knowledge and confidence in identifying, screening, and treating pediatric patients with mental health concerns and support increased engagement with family and community resources” (Institute for Health Policy and Practice, 2020). In the Spring of 2020, the UNH Project ECHO hub published the *Planning for Project ECHO® in NH: The New Hampshire Project ECHO Planning for Implementation and Business Sustainability Project Summary Report*, that included a Project ECHO needs assessment and prioritization process, that reviewed a variety of topics including pediatrics and adult behavioral health (Ryer et al., 2020). In the future, the University of New Hampshire would be well established to launch an ECHO program on adverse childhood experiences (ACEs).

Citation:

Ryer, J., West, K. M., Plante, E.-L., James, R., Miller, P., Thies, K., Doyle, M. A., White, H. C., Costello, A., Corvini, M., & Porter, J. B. (2020). *Planning for Project ECHO® in NH: The New Hampshire Project ECHO Planning for Implementation and Business Sustainability Project Summary Report*. 40.Retreived from: <https://www.citizenshealthinitiative.org/sites/default/files/media/pdfs/ECHO%20Planning%20Summary%20Report_final%20%281%29.pdf>

The **NH Prevention Community of Practice** **(*CoP*)**  was organized by NH CFEX/JSI staff. Stakeholder meetings took place every other month (funded through 2019).

Goals were to:

1) increase best practice knowledge relative to effective prevention; 2) build collegiality and professional mentoring among those delivering prevention services; 3) improve communication between and within systems of care addressing substance misuse; and 4) promote a recovery and resiliency oriented set of principles and practice constructs <https://nhcenterforexcellence.org/resources/community-of-practice-resources/2222-2/>

**Community Collaborations to Strengthen and Preserve Families** (CCSPF)

*NH DHHS & 3 local NH communities:* Manchester (Amoskeag Health).

Winnipesauke Public Health Region (Lakes Region Community Services); and

North Country (Coos Coalition)

The Community Collaborations to Strengthen and Preserve Families (CCSPF), program establishes an integrated continuum of family support, with community-based services such as mental health and substance misuse treatment, economic supports, home visiting, and educational programs, to prevent child abuse and neglect and ultimately reduce the number of children entering foster care.

*The CCSPF program goals are:*

- Reduce children entering foster care, and reduce intake and referrals to child welfare
- Increase collaboration across service systems to move towards integration and collective planning; and
- Drive future service innovations in prevention programing for children and families using data that is unique to each community. <https://www.dhhs.nh.gov/dphs/bchs/mch/ccspf.htm>

**REACT Campaign - Dartmouth Hitchcock Medical Center (DHMC)**

Dartmouth-Hitchcock’s R.E.A.C.T. Mental Health Awareness Campaign provides individuals throughout New Hampshire and Vermont with tips about how to deal with signs of emotional suffering and with resources for support and help.

The R.E.A.C.T campaign was developed to provide clear next steps to take when someone sees some, or all, of the 5 Signs of emotional suffering in another person. R.E.A.C.T supplements the 5 Signs campaign developed by Barbara Van Dahlen of Change Direction. The idea for the 5 Signs was modeled on the theory that, like knowing the 5 Signs of a heart attack or stroke, we should all know the 5 Signs of emotional distress; and when we see those signs we should take action.

Led by Dartmouth-Hitchcock Senior Director of External Affairs John Broderick, formerly the Chief Justice of the NH Supreme Court, and in collaboration with the Children’s Hospital at Dartmouth-Hitchcock, the New Hampshire Department of Education, the Vermont Agency of Education and other agencies and civic leaders across both states, Broderick has been reaching out to students, educators, parents, businesses and communities, to change the conversation on mental health and to help reduce stigma.

**Citation:**

Dartmouth Hitchcock Medical Center (DHMC), 2020. Mental Health in the Age of COVID-19 - How to "REACT". Retrieved from: <https://news.dartmouth.edu/events/event?event=60657#.X7JayWhKg2w>

**NH Mental Health Care Access in Pediatrics Project** (NHPIP/NH DHHS)

Multiyear HRSA funding:

MCAP is to promote behavioral health integration in pediatric primary care by:1) training and supporting NH's health care providers to identify and care for children with pediatric mental health conditions (Project ECHO); 2) enabling front-line health providers to receive provider teleconsultation services about the identification and treatment of children with a mental health condition; 3) enhancing the delivery of services through the creation of a referral directory of pediatric mental/behavioral health services and supports.

This project will run through September 2023. <https://www.nhpip.org/node/33>

**Trauma-informed Care in Pediatrics Project** (NHPIP)

Funded by NH Children’s Health Foundation

1) increase pediatric general practitioner knowledge about trauma-informed care and existing tools to support addressing trauma in primary care settings

2) support 4 NH pediatric primary care clinics in using quality improvement principles to pilot process(es) to detect and respond to patients experiencing toxic stress.

This project is focusing on four NH target communities/areas: Coos County, the Monadnock Region, Concord, and Nashua.

This project will run through fall 2021.

Citation:

NH Pediatric Improvement Partnership.(2020). **Trauma-informed Care in Pediatrics Project.**

<https://www.nhpip.org/node/33>

**NH Pediatric Improvement Partnership**

Citation:

NH Pediatric Improvement Partnership.(2020). Addressing Childhood Adversity and Social Determinants in Pediatric Primary Care: Recommendations for New Hampshire. Retrieved from: <https://www.nhpip.org/sites/default/files/user-uploads/NH%20ACEs%20Report%20FINAL%20July%202018.pdf>

**Preschool Development Grant** (UNH/NH Dept of Education)

The grant is a collaboration between UNH, the NH Departments of Education and Health and Human Services, and the Governor’s Council for Thriving Children, as well as New Hampshire’s early childhood advocates and practitioners.

The grant seeks to support New Hampshire’s vision that all families are afforded comprehensive and responsive supports, so they are healthy, learning, and thriving, now and in the future. The three-year (2020-2022), $26.8 million grant, will help to build New Hampshire’s early childhood care and education (ECCE) system to be effective, inclusive, responsive, efficient, and evidence-informed. The Preschool Development Grant is sponsored by the Department of Health and Human Services, Administration for Children and Families: <https://mypages.unh.edu/pdg/home>

**Interpersonal/Intrapersonal (Family and Child) Level:**

**NH Child-Parent Psychotherapy Network** (**NH Child-Parent Psychotherapy Network**  (Led by Cassie Yackley)

A collaborative network of child-parent psychotherapy (CPP) providers, trainers, and advocates. The work of the NH CPP Network has been funded by the New Hampshire Children's Health Foundation and the Project is led by Cassie Yackley, Psy.D., PLLC. The aim of the network is to connect young children and their caregivers to evidence-based intervention that addresses early exposure to adversity and trauma. <https://www.nhchildparentpsychotherapy.com/>

**Project Launch Upper Valley (DHMC)**

Funded by the Substance Abuse and Mental Health Service Administration (SAMHSA), Project LAUNCH (Linking Actions for Unmet Needs in Children’s Health) strives to promote positive behavioral health for NH’s expectant families and children through age 8. The focus is on building strong social and emotional skills for children in addition to physical and cognitive development.

By using a public health approach, Project LAUNCH is improving outcomes at the individual, family and community levels by focusing on 5 core prevention and promotion strategies:

Screening and assessment

Integration of behavioral health into primary settings

Mental health consultation in early care and education

Enhanced home visiting through increased focus on social and emotional wellbeing

Family strengthening and parent skills training.

Guided by these 5 strategies, Project LAUNCH utilizes a local-level young child wellness council and a state-level young child wellness council, both consisting of key early learning and development, health, and family support stakeholders, to develop and implement plans to improve outcomes for young children and families.

Link:

NH DHHS. 2020. Project LAUNCH. Retrieved from:

<https://sparknh.com/regional-initiatives/project-launch-manchester>

More information: [https://manchesterinklink.com/new-program-focuses-intervention-children-exposed-violence/](https://nam12.safelinks.protection.outlook.com/?url=https%3A%2F%2Fmanchesterinklink.com%2Fnew-program-focuses-intervention-children-exposed-violence%2F&data=04%7C01%7CSemra.Aytur%40unh.edu%7Cb17158464c304292bf5e08d88971e4c9%7Cd6241893512d46dc8d2bbe47e25f5666%7C0%7C0%7C637410469550013309%7CUnknown%7CTWFpbGZsb3d8eyJWIjoiMC4wLjAwMDAiLCJQIjoiV2luMzIiLCJBTiI6Ik1haWwiLCJXVCI6Mn0%3D%7C1000&sdata=mHg5qoUlE8GNo%2BaABhFWCZEzf%2BRXR10C8Fxm5gtRKfM%3D&reserved=0)

“The initiative was made possible by a three-year $150,000 grant from the HNH Foundation to Project LAUNCH (Linking Action for Unmet Needs in Children’s Health) at Manchester Community Health Center, in collaboration with the Manchester Police Department and YWCA New Hampshire.”

“The HNH Foundation was formed as the result of the merger between Blue Cross/Blue Shield of New Hampshire and Matthew Thornton Health Plan. The Foundation, incorporated in October of 1997, is organized and operates exclusively for charitable purposes. Two articles that provide a history of the HNH Foundation appeared in the New Hampshire Bar Journal.”

“Project LAUNCH (Linking Actions for Unmet Needs in Children’s Health) is funded by a federal grant by the Substance Abuse Mental Health Services Administration through the NH DHHS, Div. of Public Health Services, Maternal Child Health Section. Manchester was identified as the local community of focus and Manchester Community Health Center was chosen as the lead agency to coordinate local services and improve the systems that promote the wellness of children and families.”

**Additional References and Resources:**

ACEs Connection. “New Hampshire ACEs Initiatives and Actions.” *ACEs Initiatives and Actions*, 2017, [www.acesconnection.com/g/state-aces-action-group/fileSendAction/fcType/0/fcOid/474051083497726557/filePointer/474051083543666249/fodoid/474051083543666193/2018%20-%20New%20Hampshire.pdf](http://www.acesconnection.com/g/state-aces-action-group/fileSendAction/fcType/0/fcOid/474051083497726557/filePointer/474051083543666249/fodoid/474051083543666193/2018%20-%20New%20Hampshire.pdf)

Center for Rural Health. (2020). *Management of Opioid Use Disorder (OUD)*. <https://ruralhealth.und.edu/projects/project-echo/topics/oud-management>

Institute for Health Policy and Practice. (2020). *Project ECHO*. <https://chhs.unh.edu/institute-health-policy-practice/project-echo>

Maternal and Child Health Section, et al. “Health Indicators in Adults and Adverse Childhood Experiences (ACEs).” *NH Division of Public Health Services*, Sept. 2018, www.dhhs.nh.gov/dphs/bchs/mch/documents/nh-aces-report.pdf.

Moore, Kelly N., et al. “ACEs (Adverse Childhood Experience) Project ECHO.” *Project ECHO*, 2020, rwjms.rutgers.edu/documents/Project%20Echo/ACE%27s/ACE%27s%202020%20Curriuculum%20New%20PDF.pdf.

Metz, A., Bartley, L. (2012). Active Implementation Frameworks for Program Success: How to Use Implementation Science to Improve Outcomes for Children. National Implementation Research Network, Frank Porter Graham Child Development Institute; Chapel Hill, North Carolina

Musgrove, Ronnie, et al. “Exploring the Rural Context for Adverse Childhood Experiences (ACEs).” *National Advisory Committee on Rural Health and Human Services*, Aug. 2018, [www.hrsa.gov/sites/default/files/hrsa/advisory-committees/rural/publications/2018-Exploring-the-Rural-Context-for-ACEs.pdf](http://www.hrsa.gov/sites/default/files/hrsa/advisory-committees/rural/publications/2018-Exploring-the-Rural-Context-for-ACEs.pdf).

NAMI New Hampshire, State Suicide Prevention Council (SPC) and Youth Suicide Prevention Assembly (YSPA).(2018). New Hampshire Suicide Prevention Annual Report. Retrieved from: <https://www.dhhs.nh.gov/dphs/bchs/spc/documents/2018-spc-annual-report.pdf>

Ports, Katie A., et al. “Adverse Childhood Experiences and Suicide Risk: Toward Comprehensive Prevention.” *American Journal of Preventative Medicine*, 5 May 2017, www.ajpmonline.org/article/S0749-3797(17)30205-2/fulltext.

Rutgers, The State University of New Jersey. (2018). *Adverse Childhood Experiences*. <https://rwjms.rutgers.edu/community_health/other/project-echo/adverse-childhood-experiences>

Schofield, Thomas J., et al. “Intergenerational Continuity in Adverse Childhood Experiences and Rural Community Environments.” *AJPH CHILD MALTREATMENT*, Sept. 2018, ajph.aphapublications.org/doi/pdfplus/10.2105/AJPH.2018.304598.

SparkNH. “Understanding Adverse Childhood Experiences (ACEs).” *Domain*, 2018, sparknh.com/news/understanding-adverse-childhood-experiences-aces/.

Stillman, Laurie R. “A Community Comes Together to Protect Its Children: Adverse Childhood Experience Response Team (ACERT) in Manchester, New Hampshire.” *New Hampshire Children's Health Foundation*, Sept. 2019, nhchildrenshealthfoundation.org/assets/2019/10/2019-ACERT-Case-Study.pdf.

Substance Abuse and Mental Health Services Administration. (2019). Key Substance Use and Mental Health Indicators in the United States: Results from the 2018 National Survey on Drug Use and Health. Retrieved from: <https://www.samhsa.gov/data/sites/default/files/cbhsq-reports/NSDUHNationalFindingsReport2018/NSDUHNationalFindingsReport2018.pdf>

The University of New Mexico. (2020a). *Our Story*. <https://hsc.unm.edu/echo/about-us/>

The University of New Mexico. (2020b). *Project ECHO*. <https://hsc.unm.edu/echo/>
