# Supplementary material for: Social‐ecological theory, substance misuse, adverse childhood experiences, and adolescent suicidal ideation: Applications for community–academic partnerships
Source: J Community Psychol. 2021 May 4;50(1):265–84. doi: 10.1002/jcop.22560 (PMC9292564; doi:10.1002/jcop.22560)
Supplement: Supplementary file 2 — Supporting information. [file JCOP-50-265-s002.rtf]

FEDERAL


STATE
New Future's Children's Behavioral Health Program
